# Poisoning substances taken by young people: a population-based cohort study

**DOI:** 10.3399/bjgp18X698897

**Published:** 2018-09-11

**Authors:** Edward G Tyrrell, Denise Kendrick, Kapil Sayal, Elizabeth Orton

**Affiliations:** Division of Primary Care;; Division of Primary Care;; Division of Psychiatry & Applied Psychiatry;; Division of Primary Care, School of Medicine, University of Nottingham, Nottingham.

**Keywords:** adolescent, general practice, poisoning, self-injurious behavior, young adult

## Abstract

**Background:**

Globally, poisonings account for most medically-attended self-harm. Recent data on poisoning substances are lacking, but are needed to inform self-harm prevention.

**Aim:**

To assess poisoning substance patterns and trends among 10–24-year-olds across England

**Design and setting:**

Open cohort study of 1 736 527 young people, using linked Clinical Practice Research Datalink, Hospital Episode Statistics, and Office for National Statistics mortality data, from 1998 to 2014.

**Method:**

Poisoning substances were identified by ICD-10 or Read Codes. Incidence rates and adjusted incidence rate ratios (aIRR) were calculated for poisoning substances by age, sex, index of multiple deprivation, and calendar year.

**Results:**

In total, 40 333 poisoning episodes were identified, with 57.8% specifying the substances involved. The most common substances were paracetamol (39.8%), alcohol (32.7%), non-steroidal anti-inflammatory drugs (NSAIDs) (11.6%), antidepressants (10.2%), and opioids (7.6%). Poisoning rates were highest at ages 16–18 years for females and 19–24 years for males. Opioid poisonings increased fivefold from 1998–2014 (females: aIRR 5.30, 95% confidence interval (CI) = 4.08 to 6.89; males: aIRR 5.11, 95% CI = 3.37 to 7.76), antidepressant poisonings three-to fourfold (females: aIRR 3.91, 95% CI = 3.18 to 4.80, males: aIRR 2.70, 95% CI = 2.04 to 3.58), aspirin/NSAID poisonings threefold (females: aIRR 2.84, 95% CI = 2.40 to 3.36, males: aIRR 2.76, 95% CI = 2.05 to 3.72) and paracetamol poisonings threefold in females (aIRR 2.87, 95% CI = 2.58 to 3.20). Across all substances poisoning incidence was higher in more disadvantaged groups, with the strongest gradient for opioid poisonings among males (aIRR 3.46, 95% CI = 2.24 to 5.36).

**Conclusion:**

It is important that GPs raise awareness with families of the substances young people use to self-harm, especially the common use of over-the-counter medications. Quantities of medication prescribed to young people at risk of self-harm and their families should be limited, particularly analgesics and antidepressants.

## INTRODUCTION

Poisonings among young people are an important but potentially avoidable cause of morbidity and mortality.^1^^,^^2^ They are one of the leading causes of death at this age, including some unintentional, but primarily self-harm events.^3^^,^^4^ In the UK, self-harm affects up to 1 in 7 young people^5^^–^7 with poisonings accounting for 77–91% of all medically-attended self-harm episodes.^8^^–^10 Young people who have self-poisoned are 10–32 times more likely to complete suicide over the next 10 years than those who have not.^11^ As outlined in the national Suicide Prevention Strategy, primary care has a key role to play in managing those at risk of self-harm.^12^ Up-to-date information on the substances involved in poisonings among young people is needed to guide prescribing decisions as well as the advice offered to individuals at risk and their families. However, at a population level in the UK, these data are lacking.

Young people represent a distinct population in terms of risk of self-harm and clinician prescribing practices. Most UK data describing poisoning substances among young people have been based on attendances at specific hospitals or emergency departments (EDs) with limited generalisability,^8^^,^^9^^,^^13^ have combined data on young people and adults,^14^^–^16 or are now out of date.^17^ International data are now largely out of date;^18^^–^23 however, two US studies have described poisoning substances in young people presenting to services up to 2013,^24^^,^^25^ one of which solely examined opiate poisonings.^25^

This study aimed to estimate population level medically-attended poisoning incidence among 10–24-year-olds in England between 1998 and 2014 according to poisoning substance by sex, age, deprivation level, and changes over time.

## METHOD

### Study population

This study utilised routinely collected General Practice data from the Clinical Practice Research Datalink (CPRD). In the UK, 98% of the population is registered with a GP,^26^ and primary care records include information on all healthcare attendances. CPRD includes anonymised medical records from, at the point of data extraction, 674 UK general practices, 395 of which had health records linked to Hospital Episode Statistics (HES) data (recording hospital admissions in England), and Office for National Statistics (ONS) mortality data. Using these three linked data sources improves poisoning ascertainment compared to CPRD or HES data alone.^27^^,^^28^ The study population was an open cohort of young people registered with a HES–ONS linked CPRD practice and aged 10–24 years between 1 April 1998 and 31 March 2014. This cohort has been described in detail elsewhere.^29^

**How this fits in**

Poisonings are by far the most common form of medically-attended self-harm among young people and are often avoidable, but up-to-date information on the substances young people are being poisoned by is lacking. This study shows the most common substances involved in poisonings among 10–24-year-olds in England are paracetamol, alcohol, NSAIDs, antidepressants, and opioids. Between 1998 and 2014 there was a fivefold increase in opioid poisonings among 10–24-year-olds, as well as a three- to fourfold increase in antidepressant poisonings. Poisonings show a close relationship with deprivation, with the incidence of poisoning from all substances rising with increasing socioeconomic deprivation.

### Outcome events

**Figure 1.**
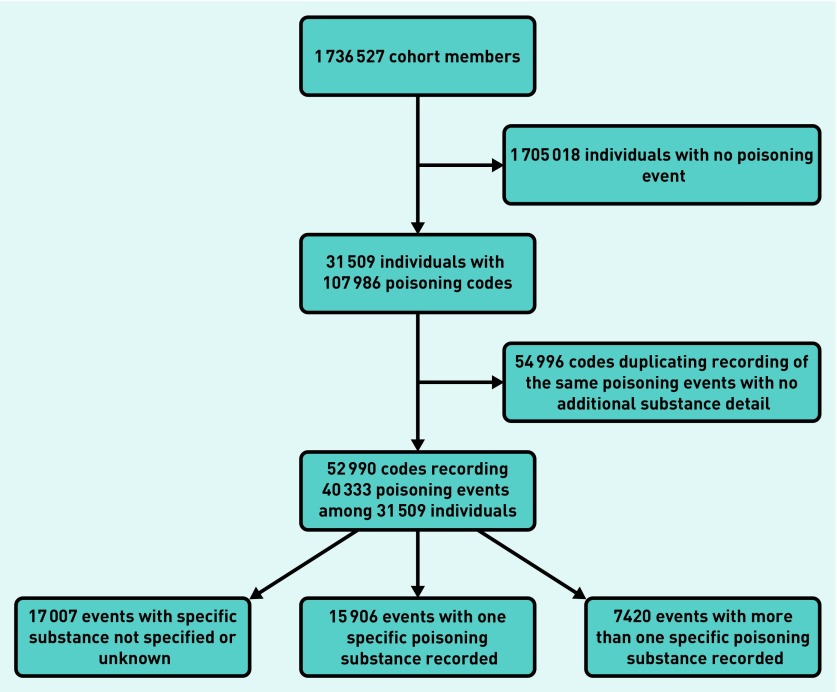
***Number of poisoning codes and events.***

The study outcome was a poisoning event occurring within the study period and recorded within any one of the three linked data sources. Repeat poisoning events within the same individual were included. Time dependent algorithms that have been previously developed^27^ excluded duplicate codes recording the same poisoning events (Figure 1). For ONS mortality data, only primary cause of death codes were included.

Poisonings, and their intent, were identified using a combination of International Classification of Diseases (ICD)-10 codes (T36–65, X40–49, X60–69, Y10–19, and Y90–91) in HES/ONS data and Read Codes in CPRD data. Poisoning substances were categorised using ICD-10 categories T35–65, excluding venomous animal and food poisonings (T61, T63, and T64). The comprehensive poisoning code list used has been described elsewhere.^29^ All substances recorded for a poisoning event have been included. Therefore, one poisoning event may have had more than one contributing substance.

### Statistical analysis

The number and percentage of poisoning events involving specific substances was identified. Incidence rates (IR) per 100 000 person-years (PY) and 95% confidence intervals (95% CI) were calculated by sex, age, socioeconomic deprivation, geographical area, and calendar year for the five most common substances, as well unspecified substance poisonings and total poisonings. For clinical relevance, poisonings involving non-steroidal anti-inflammatory drugs (NSAIDs) (T39.3) and aspirin (T39.0) were combined for calculating incidence rates, as were all antidepressants (T43.0–T43.2). Age and year were included as time-varying covariates by Lexis expansion. Time period was analysed in single years in the univariate analysis and 4-year bands in the multivariate analysis. Socioeconomic status was assessed using quintiles of the Index of Multiple Deprivation 2010 (IMD) for England. This was provided by CPRD, at a lower super output area level (approximately 650 households), based on the postcode of the individual’s GP practice.

Adjusted incidence rate ratios (aIRR) were calculated for the five most common substances, total poisonings (regardless of whether an exact substance was recorded for the poisoning or not) and unspecified substance poisonings using negative binomial regression, and adjusted for each of the covariates outlined above. Potential interactions were assessed including sex and age,^9^ time and sex,^9^ time and age,^20^ time and socioeconomic deprivation, with *P*<0.01 taken as significant. Where significant interactions were found, stratified incidence rates are presented. Data were analysed using Stata/SE (version 13).

## RESULTS

The cohort contained 1 736 527 young people contributing 7 209 529 person-years of follow-up: 52% were female (Table 1). In total, 40 333 poisoning events were recorded from 31 509 young people. Of those, 23 326 (57.8%) had poisoning substance(s) recorded. One-third (31.8%) had more than one poisoning substance recorded (Figure 1). Two-thirds (66.5%) of all poisonings were recorded as intentional, 7.5% unintentional, and 26.0% of undetermined or unspecified intent.

**Table 1.** Characteristics of the cohort

**Characteristics**	**Participants, *n* (%), *N* = 1 736 527**	

	**No poisoning events, *N* = 1 705 018**	**At least one poisoning event, *N*= 31 509**
**Sex**		
Male	812 770 (48)	12 345 (39)
Female	892 248 (52)	19 164 (61)

**Median age, years, (IQR) entering cohort**	16.3 (10.0–21.5)	13.9 (10.0–18.5)

**Median person-years (IQR) contributed**	2.8 (1.1–6.3)	6.7 (3.2–10.2)

**Index of Multiple Deprivation quintile**		
1 (least deprived)	231 391 (14)	3021 (10)
2	362 948 (21)	5834 (18)
3	340 261 (20)	6867 (22)
4	392 703 (23)	7826 (25)
5 (most deprived)	377 715 (22)	7961 (25)

IQR = interquartile range.

### Most common substances

Paracetamol was the most commonly involved substance (39.8%, Table 2). The other most common substances were ethanol (drinking alcohol) (32.7%), NSAIDs (11.6%), other/unspecified antidepressants (includes selective serotonin reuptake inhibitors [SSRIs] and serotonin and norepinephrine reuptake inhibitors [SNRIs]) (10.2%), other opioids (includes codeine, tramadol, and morphine) (7.6%), benzodiazepines (4.6%), aspirin (4.6%), psychostimulants (includes amphetamines, methylphenidate, and ecstasy) (4.2%), other antiepileptic/sedative hypnotic drugs (includes Z-drugs, such as zopiclone) (3.2%), and tricyclic/tetracyclic antidepressants (2.7%).

**Table 2.** Ten most common poisoning substances by five character ICD-10 code

**Substance (by 5 character ICD-10 code)**	**Poisoning events including named substance, *n***	**Events involving substance, % (*N* = 23 326)**
T39.1 4-Aminophenol derivatives *(paracetamol)*	9289	39.8
T51.0 Ethanol *(drinking alcohol)*	7635	32.7
T39.3 Other non-steroidal anti-inflammatory drugs [NSAIDs]	2696	11.6
T43.2 Other and unspecified antidepressants *(includes SSRIs)*	2373	10.2
T40.2 Other opioids	1766	7.6
T42.4 Benzodiazepines	1075	4.6
T39.0 Salicylates *(aspirin)*	1072	4.6
T43.6 Psychostimulants with abuse potential	976	4.2
T42.6 Other antiepileptic and sedative hypnotic drugs	736	3.2
T43.0 Tricyclic and tetracyclic antidepressants	624	2.7

Italics not included in ICD-10 description but added by the authors for explanation. ICD = International Classification of Diseases. SSRIs = selective serotonin reuptake inhibitors.

### Age and sex variations

**Figure 2.**
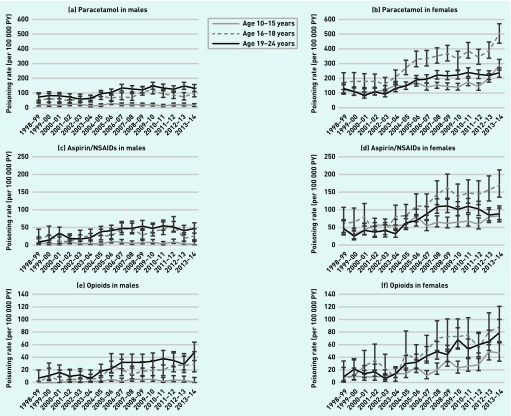

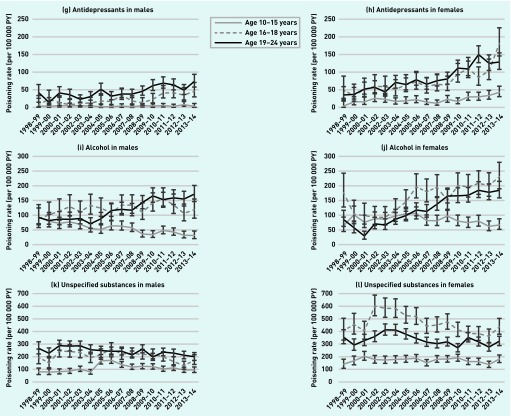
***Incidence rates by age over time for a) poisonings involving paracetamol in males, b) poisonings involving paracetamol in females, c) poisonings involving aspirin/NSAIDs in males, d) poisonings involving aspirin/NSAIDs in females, e) poisonings involving opioids in males, f) poisonings involving opioids in females. g) poisonings involving antidepressants in males, h) poisonings involving antidepressants in females, i) poisonings involving alcohol in males, j) poisonings involving alcohol in females, k) unspecified substance poisonings in males, l) unspecified substance poisonings in females.***

Poisoning incidence according to age varied between males and females (Figure 2) (*P*<0.001 for each substance). The peak poisoning incidence in females was in the 16–18-year age group for all substances other than antidepressants (for example, aIRR 2.55, 95% CI = 2.12 to 3.07 for 16–18-year-olds compared to 10–15-year-olds for opioid poisonings, Table 3). The peak poisoning incidence in males was in the oldest age group for all substances (for example, aIRR 8.68, 95% CI = 6.68 to 11.28 for 19–24-year-olds compared to 10–15-year-olds for aspirin/NSAID poisonings).

**Table 3.** Adjusted poisoning incidence rate ratios for five most common substances, total poisonings and unspecified/unknown substance poisonings

	**Adjusted poisoning incidence rate ratios (95% CIs)**						

	**All poisonings combined (*n*= 40 333)**	**Paracetamol (T39.1) (*n*= 9 289)**	**Aspirin/NSAIDs (T39.0 & T39.3) (*n*= 3 618)**	**Opioids (T40.2) (*n*= 1 766)**	**Antidepressants (T43.0, T43.1, T43.2) (*n*= 2 945)**	**Alcohol (T51.0) (*n*= 7 635)**	**Unspecified/unknown substance (*n*= 17 007)**
**Females**							

**Age group, years**							
10–15	1.00	1.00	1.00	1.00	1.00	1.00	1.00
16–18	2.60 (2.49 to 2.72)	2.40 (2.20 to 2.61)	2.13 (1.87 to 2.43)	2.55 (2.12 to 3.07)	4.73 (3.93 to 5.69)	2.19 (1.98 to 2.44)	3.39 (3.16 to 3.63)
19–24	1.83 (1.76 to 1.91)	1.33 (1.23 to 1.43)	1.42 (1.26 to 1.78)	2.11 (1.78 to 2.49)	6.17 (5.23 to 7.28)	1.73 (1.58 to 1.90)	2.62 (2.46 to 2.80)

**Calendar period**							
1998/99–2001/02	1.00	1.00	1.00	1.00	1.00	1.00	1.00
2002/03–2005/06	1.20 (1.14 to 1.27)	1.52 (1.36 to 1.70)	1.49 (1.25 to 1.78)	1.56 (1.19 to 2.04)	1.46 (1.18 to 1.82)	1.43 (1.26 to 1.64)	1.12 (1.03 to 1.21)
2006/07–2009/10	1.25 (1.19 to 1.32)	2.16 (1.94 to 2.41)	2.51 (2.13 to 2.97)	3.77 (2.90 to 4.90)	1.79 (1.45 to 2.21)	1.88 (1.65 to 2.13)	0.89 (0.82 to 0.96)
2010/11–2013/14	1.48 (1.40 to 1.56)	2.87 (2.58 to 3.20)	2.84 (2.40 to 3.36)	5.30 (4.08 to 6.89)	3.91 (3.18 to 4.80)	2.24 (1.97 to 2.55)	0.88 (0.81 to 0.95)

**IMD quintile**							
1 (least deprived)	1.00	1.00	1.00	1.00	1.00	1.00	1.00
2	1.50 (1.41 to 1.60)	1.33 (1.18 to 1.50)	1.30 (1.08 to 1.56)	1.56 (1.19 to 2.04)	2.00 (1.57 to 2.54)	1.55 (1.34 to 1.80)	1.51 (1.37 to 1.66)
3	1.76 (1.65 to 1.88)	1.40 (1.24 to 1.59)	1.20 (1.00 to 1.45)	1.58 (1.20 to 2.09)	1.63 (1.27 to 2.08)	1.65 (1.42 to 1.92)	2.21 (2.00 to 2.43)
4	2.02 (1.90 to 2.15)	1.60 (1.42 to 1.81)	1.47 (1.22 to 1.76)	1.70 (1.31 to 2.22)	2.49 (1.96 to 3.17)	1.98 (1.71 to 2.29)	2.37 (2.15 to 2.61)
5 (most deprived)	2.02 (1.90 to 2.16)	1.66 (1.47 to 1.88)	1.52 (1.26 to 1.76)	2.12 (1.63 to 2.76)	2.29 (1.80 to 2.92)	1.82 (1.57 to 2.11)	2.23 (2.02 to 2.46)

**Males**							

**Age group, years**							
10–15	1.00	1.00	1.00	1.00	1.00	1.00	1.00
16–18	2.48 (2.34 to 2.62)	4.59 (3.86 to 5.31)	6.52 (4.92 to 8.63)	7.19 (4.83 to 10.71)	7.26 (5.23 to 10.08)	2.36 (2.12 to 2.62)	2.02 (1.87 to 2.19)
19–24	3.17 (3.01 to 3.34)	7.12 (6.15 to 8.24)	8.68 (6.68 to 11.28) 13.02 (8.94 to 18.96)		18.56 (13.67 to 25.21)	2.37 (2.15 to 2.61)	2.48 (2.30 to 2.67)

**Calendar period**							
1998/99–2001/02	1.00	1.00	1.00	1.00	1.00	1.00	1.00
2002/03–2005/06	1.03 (0.96 to 1.09)	1.16 (0.98 to 1.37)	1.62 (1.19 to 2.20)	1.87 (1.20 to 2.93)	1.05 (0.78 to 1.43)	0.97 (0.86 to 1.10)	1.02 (0.94 to 1.11)
2006/07–2009/10	1.06 (1.00 to 1.12)	1.81 (1.55 to 2.12)	2.85 (2.13 to 3.82)	4.17 (2.75 to 6.32)	1.51 (1.13 to 2.01)	1.15 (1.02 to 1.29)	0.93 (0.86 to 1.01)
2010/11–2013/14	1.01 (0.95 to 1.07)	1.79 (1.53 to 2.10)	2.76 (2.05 to 3.72)	5.11 (3.37 to 7.76)	2.70 (2.04 to 3.58)	1.24 (1.10 to 1.39)	0.79 (0.72 to 0.86)

**IMD quintile**							
1 (least deprived)	1.00	1.00	1.00	1.00	1.00	1.00	1.00
2	1.62 (1.50 to 1.75)	1.49 (1.22 to 1.81)	1.80 (1.30 to 2.49)	1.86 (1.18 to 2.94)	1.89 (1.32 to 2.72)	1.34 (1.16 to 1.55)	1.86 (1.65 to 2.10)
3	2.51 (2.32 to 2.72)	1.35 (1.10 to 1.65)	1.47 (1.05 to 2.07)	1.72 (1.07 to 2.74)	1.98 (1.37 to 2.86)	1.61 (1.39 to 1.87)	4.30 (3.83 to 4.82)
4	2.66 (2.46 to 2.88)	1.63 (1.34 to 1.97)	1.73 (1.24 to 2.41)	2.22 (1.41 to 3.48)	2.24 (1.57 to 3.21)	1.68 (1.45 to 1.94)	4.63 (4.13 to 5.20)
5 (most deprived)	2.62 (2.42 to 2.83)	2.16 (1.79 to 2.62)	1.94 (1.40 to 2.70)	3.46 (2.24 to 5.36)	2.82 (1.97 to 4.02)	1.67 (1.45 to 1.93)	3.90 (3.45 to 4.40)

All mutually adjusted for age, calendar period, IMD and Area of England. IMD = Index of Multiple Deprivation.

### Changes over time

Poisonings increased more over time among females than males for each of the five most common substances (Figure 2). For paracetamol, aspirin/NSAID, opioid, and antidepressant poisonings this was most marked among 16–18-year-old females (for example, paracetamol poisonings, IR 171.6 per 100 000 person-years (PY), 95% CI = 123.8 to 237.9 in 1998–1999 to 501.3 per 100 000 PY, 95% CI = 439.1 to 571.6 in 2013–2014). There was a sharp rise in paracetamol poisonings among 10–15-year-old females in the most recent 2 years (IR 142.3 per 100 000 PY, 95% CI = 120.4 to 168.3 in 2011–2012 to 290.0, 95% CI = 255.6 to 329.1 in 2013–2014). For poisonings involving alcohol, the biggest increase was among 19–24-year-old females (IR 85.4 per 100 000 PY, 95% CI = 62.1 to 117.4 in 1998–1999 to 185.2, 95% CI = 159.2 to 215.4 in 2013–2014).

Though smaller than among females, the increases seen over time among males were largest in the 19–24-year age group for each of the five most common substances. The biggest of these increases was for opioid poisonings (IR 6.6 per 100 000 PY, 95% CI = 2.1 to 20.3 in 1998–1999 to 47.6, 95% CI = 35.3 to 64.2 in 2013–2014).

Poisoning events involving alcohol among 10–14-year-old males decreased over time (IR 94.4 per 100 000 PY, 95% CI = 71.4 to 124.9 in 1998–1999 to 31.2, 95% CI = 21.4 to 45.5 in 2013–2014). There was also a reduction in unspecified substance poisonings among 16–18-year-old females from their peak in 2001–2002 (IR 601.8 per 100 000 PY, 95% CI = 525.5 to 689.2) to 2013–2014 (437.6 per 100 000 PY, 95% CI = 380.0 to 503.9)

After adjusting for available confounders, poisonings involving the five most common substances all increased steadily between 1998 and 2014 in both sexes. The largest increases saw opioid poisonings increase fivefold from 1998–2014 (females: aIRR 5.30, 95% CI = 4.08 to 6.89, males: aIRR 5.11, 95% CI = 3.37 to 7.76), antidepressant poisonings three- to fourfold (females: aIRR 3.91, 95% CI = 3.18 to 4.80, males: aIRR 2.70, 95% CI = 2.04 to 3.58), aspirin/NSAID poisonings threefold (females: aIRR 2.84, 95% CI = 2.40 to 3.36, males: 2.76, 95% CI = 2.05 to 3.72), and paracetamol poisonings threefold in females (aIRR 2.87, 95% CI = 2.58 to 3.20). Unspecified substance poisonings decreased over time in both sexes, by 12% in females (aIRR 0.88, 95% CI = 0.81 to 0.95) and 21% in males (aIRR 0.79, 95% CI = 0.72 to 0.86) (Table 3).

### Socioeconomic variations

A social gradient in poisonings was demonstrated for each substance with a roughly twofold increase in poisoning incidence for the most compared to least deprived groups. Excluding unspecified poisonings, the steepest gradient was for opioid poisonings in males with a 3.5 fold increase in poisoning incidence in the most compared to least deprived group (aIRR 3.46, 95% CI = 2.24 to 5.36) (Table 3).

## DISCUSSION

### Summary

The most common substances involved in poisonings among 10–24-year-olds were paracetamol, alcohol, NSAIDs, antidepressants, and opioids. There were increases in poisoning incidence rates of between three- and fivefold for opioids, antidepressants, aspirin/NSAIDs and paracetamol over the study period. Sex and age variation was shown with the largest rises over time being in 16–18-year-olds among females and 19–24-year-olds among males. There was a social gradient in poisonings for each of the five most common substances, with higher poisoning incidence in the most deprived groups.

### Strengths and limitations

This is the first study to use population level combined English healthcare data sources to examine the substances involved in poisoning episodes among young people. As CPRD has been shown to be broadly representative of the UK population,^30^ these results should be generalisable to the wider UK and other similar countries. This study is among the largest to examine poisoning substances in young people worldwide. The use of three linked databases should have resulted in greater ascertainment of poisoning incidence than studies using single data sources from localised areas or using CPRD or HES data alone.^27^^,^^28^

Limitations of this study include underestimation of poisoning incidence for specific substances as only 57.8% of the poisonings had substances involved recorded. These results showed a reduction in unspecified or unknown substance poisonings over time, but as the absolute rate reductions among unspecified or unknown poisonings were much smaller than the absolute rate increases seen for the five most common substances, improved recording over time cannot fully explain the incidence rate increases found for these substances. It was only possible to obtain deprivation data based on the IMD score of an individual’s GP practice address, which may not always accurately represent an individual household’s deprivation level. Finally, because of the large sample size some statistically significant results that are not clinically important may have been detected.

### Comparison with existing literature

The finding that the most common substances involved in poisoning were over-the-counter (OTC) analgesics is consistent with previous studies from the UK,^8^^,^^9^^,^^13^^,^^31^ US,^18^^,^^24^ and Australia.^32^ In the current findings, alcohol was the second most common substance involved, similar to findings among comparable age groups in Finland,^20^ Norway,^22^ the US,^18^ and emergency department (ED) attendances for any form of self-harm among 12–25-year-olds in Leeds, England.^8^

The most up-to-date UK poisoning substance data comes from the Multicentre Study of Self-harm, reporting ED data from five hospitals across three English cities from 2003–2012 in people aged >15 years. This showed 46% of poisonings involved paracetamol or salicylates, 25% antidepressants, 14% benzodiazepines, and 7% major tranquilisers and/or antipsychotics.^16^ Though there are similarities with the most common substances demonstrated in the current study, the most notable difference is the presence of opioids among the most common substances reported here, suggesting they may be a more common poisoning substance among young people than other adults.

Though the authors are not aware of recent data on temporal trends in opioid poisonings in the UK, data from US national hospital discharges show almost doubling of opioid poisonings among 15–19-year-olds between 1997 and 2012.^25^ The current results suggest this increase may be larger among 10–24-year-olds in the UK, with a fivefold increase in opioid poisonings over a similar time period. One potential explanation may be the increasing opioid prescribing rates in the UK. Among >18-year-olds prescriptions for weak opioids nearly doubled from 2005–2012,^33^ while prescriptions of strong opioids increased four- to sixfold from 2000–2012.^33^^,^^34^ GPs face a number of challenges around opioid prescribing including evidence of limited long-term benefits for chronic pain,^35^^,^^36^ alongside often having ethical concerns about withholding analgesia.^37^ Such consultations are frequently set in the context of patients fearful of an impact on pain management, their quality of life or possible dependence. However, there is evidence that reducing access to lethal means, including medication, reduces suicides.^2^

Limiting prescribing is not the only possible approach to reducing poisonings. Educating physicians and potentially the public is key, with physician education in suicide prevention having been shown to reduce suicide rates.^2^ There is also evidence that legislation and regulations, such as that introduced in the UK in 1998 to limit paracetamol pack sizes sold over-the-counter, reduces poisoning deaths.^1^

The finding of a three- to fourfold increase in antidepressant poisonings from 1998–2014 continues the temporal trend among 12–18 year-olds shown by Hawton *et al*, who demonstrated a more than doubling in antidepressant poisonings from 1996–2000.^9^ US data showed a near doubling of antidepressant poisonings in 13–19-year-olds between 2004 and 2012.^24^ Increases in antidepressant poisonings may again reflect increased UK prescribing. There was a 28% increase in antidepressant prescribing among 6–18-year-olds from 2003–2013 in Wales,^38^ and a doubling of antidepressant prescriptions among >14-year-olds in the UK from 1995–2011.^39^ However, this should be considered in light of the fact that higher antidepressant prescription rates are actually associated with decreasing suicide rates through their benefits in treating depression itself.^2^

In the UK, paracetamol poisonings increased during the 1990s but reduced around 1998–1999,^9^^,^^17^ most likely due to legislation restricting the pack size of paracetamol. There is some evidence of further increases in the early 2000s.^9^^,^^13^ The current study suggests continued increases in paracetamol poisoning since then. US data, in contrast, shows static paracetamol poisoning levels among 13–19-year-olds between 2004 and 2013.^24^

The threefold increase in aspirin/NSAID poisonings demonstrated updates earlier UK evidence of a more than halving in NSAID poisonings among 12–18-year-olds between 1978–1990 and 1991–2003 and static rates in <15-year-olds from 1990–2000.^9^

The overall increase in poisonings involving alcohol is similar to previous findings from UK primary care data that reported a 20% increase in alcohol poisonings among 10–17-year-olds between 1992 and 2012.^40^ The greater increase among females compared to males and the reduction in 10–15-year-old male alcohol poisonings reflect national trends in underage binge drinking that have increased from 1995–2011 among girls but fallen slightly among boys.^41^

The findings of a social gradient in poisonings among young people reflect similar findings of 1.5 to threefold increased risk in the most compared to least deprived groups shown across a variety of countries and settings.^10^^,^^40^^,^^42^^–^45 To the authors’ knowledge, this social gradient has not previously been quantified for individual substances. This gradient may reflect differences in psychological distress, psychosocial stressors, and support^46^ as well as varied social, economic, and physical environments across the different socioeconomic groups.^47^

### Implications for research and practice

Future research to examine poisoning incidence in conjunction with individual prescription data would be useful to describe the source of poisonings substances used by young people.

Alongside physician education, these findings primarily suggest volumes of medication prescribed to young people should be limited. This is especially relevant for those with a history of self-harm, where similar precautions should be taken when prescribing for family members. This is important when prescribing for both psychiatric and non-psychiatric conditions, especially analgesics. Though the largest increases in poisonings were seen for opioids, adding to the increasing volume of recent evidence suggesting the need to curb opioid prescribing, this should be seen in the context of the most common poisoning substances being OTC analgesics. It is important for GPs, as well as hospital-based clinicians assessing young people after an episode of self-harm, to raise awareness of the types of substances young people use to self-harm with the families of those at risk. In particular, that these commonly include OTC medications that may be easily accessible within households or which young people may buy themselves. This is also a key message for community pharmacists, when selling over-the-counter analgesia to young people.
